# A Hypothesis on the Progression of Insulin-Induced Lipohypertrophy: An Integrated Result of High-Frequency Ultrasound Imaging and Blood Glucose Control of Patients

**DOI:** 10.3390/diagnostics13091515

**Published:** 2023-04-23

**Authors:** Jian Yu, Hong Wang, Meijing Zhou, Min Zhu, Jing Hang, Min Shen, Xin Jin, Yun Shi, Jingjing Xu, Tao Yang

**Affiliations:** 1 Department of Endocrinology, The First Affiliated Hospital with Nanjing Medical University (Jiangsu Province Hospital), Nanjing 210029, China; 2 Department of Ultrasound, The First Affiliated Hospital with Nanjing Medical University (Jiangsu Province Hospital), Nanjing 210029, China; 3 Department of Hospital Pharmacy, The Affiliated Suqian First People’s Hospital of Nanjing Medical University, Suqian 223800, China; 4 Department of Nursing, The First Affiliated Hospital with Nanjing Medical University (Jiangsu Province Hospital), Nanjing 210029, China

**Keywords:** lipohypertrophy (LH), glycated hemoglobin (HbA_1C_), ultrasound, insulin injection

## Abstract

Aims: To put forward a scientific hypothesis about the progression of insulin-injection-induced lipohypertrophy (LH) according to the high-frequency ultrasonic imaging of insulin injection sites and the blood glucose control of patients. Methods: A total of 344 patients were screened for LH by means of high-frequency ultrasound scanning. The results of their ultrasound examination were described in detail and categorized into several subtypes. Seventeen patients with different subtypes of LH were followed up to predict the progression of LH. To further verify our hypothesis, the effects of different types of LH on glycemic control of patients were observed by comparing glycated hemoglobin A1c (HbA_1C_) and other glycemic-related indicators. Results: LH was found in 255 (74.1%) patients. According to the high-frequency ultrasonic imaging characteristics, LH can be categorized into three subtypes in general. Among all the LHs, the most common type observed was nodular hyperechoic LH (*n* = 167, 65.5%), followed by diffuse hyperechoic LH (*n* = 70, 27.5%), then hypoechoic LH (*n* = 18, 7.0%). At the follow-up after six months, all 10 patients with nodular hyperechoic LH had LH faded away. Of the five patients with diffuse hyperechoic LH, two had inapparent LH, and three had diffuse hyperechoic parts which had shrunk under ultrasound. No obvious changes were observed in the two cases of hypoechoic LH. Compared with the LH-free group, the mean HbA_1C_ of the nodular hyperechoic LH group increased by 0.8% (9 mmol/mol) (95% CI:−1.394~−0.168, *p* = 0.005), that of the diffuse hyperechoic LH group increased by 2.0% (21 mmol/mol) (95% CI: −2.696~−1.20, *p* < 0.001), and that of the hypoechoic LH group increased by 1.5% (16 mmol/mol) (95% CI: −2.689~−0.275, *p* = 0.007). Conclusions: It was hypothesized that the earlier stage of LH is nodular hyperechoic LH. If nodular LH is not found in time and the patient continues to inject insulin at the LH site and/or reuse needles, LH will develop into a diffuse type or, even worse, a hypoechoic one. Different subtypes of LH may represent differences in severity when blood glucose control is considered as an important resolution indicator. Further studies are needed to confirm our hypothesis on the progression and reversion of insulin-induced lipohypertrophy.

## 1. Introduction

The number of patients with diabetes worldwide is estimated to reach 783 million by 2045 [1]. All patients with type 1 diabetes and type 2 diabetes who are not effective at controlling their blood glucose with oral medications will have to initiate insulin injection therapy eventually. So, a considerable number of patients with diabetes need insulin therapy to maintain their glucose at a near-normal level. Lipohypertrophy (LH) is the most common local complication caused by multiple overlapping insulin injections, and it is one of the most prominent contributors to poor metabolic control [2]. Moreover, LH is associated with nearly one-third greater insulin consumption, with large cost implications [3].

The existing methods for detecting insulin injection-related lipohypertrophy (LH) mainly include two techniques—clinical examination (inspection and palpation) and high-frequency ultrasound scanning. Clinical examination tends to be more convenient and cost-effective, whereas ultrasonography can provide us with a further understanding of LH, especially in terms of different pathological changes related to LH. After the concept of subclinical LH was proposed [4,5], ultrasound scanning was proposed as a potentially more advanced and objective method for detecting LH because ultrasound can better describe the characteristics and morphology of LH induced by insulin injection [6,7] and can rather effectively reduce the rate of missed diagnosis of LH [8,9].

LH has received much attention in the past decade because it has previously been observed that the occurrence of LH is associated with poorer glycemic control in patients, regardless of whether the diagnosis of LH in these studies was based on clinical examination or ultrasound screening [8,10,11,12]. Moreover, Volkova [4] confirmed that LH detected by ultrasonography without visual and palpable changes could also worsen the compensation of glycemic control, which further emphasizes the importance of using ultrasound scanning in detecting LH.

Overall, these studies focused on identifying the presence or absence of LH and evaluating the relationship between the occurrence of LH and glycemic control. To go further, there is little discussion about whether there are different types of LH and whether the presence of LH (no matter which type) will definitely affect the control of blood glucose of patients.

Currently, our understanding of LH is still far from comprehensive, especially regarding the classification or development process of LH. We firmly believe that, like all other diabetes-related complications, we need to explore the progression of LH in depth and try to grade LH scientifically, so that more targeted treatment or prevention measures can be taken.

Clinically, ultrasound has been of great value in assessing the severity of many diseases for a long time [13,14]. Therefore, the main purpose of this study is to screen all insulin injection sites of patients by means of high-frequency ultrasound and to find out whether patients have LH and summarize the type of LH. The changes in the ultrasound images of those existing LHs after stopping the injection at the lesion site were followed up. Then, we propose a scientific hypothesis about the progression of LH. On this basis, glycated hemoglobin (HbA1c), TIR (time in range) and CV (coefficient of variation), as the key indicators of blood glucose control of patients with different subtypes of LH, are compared to verify our hypothesis.

## 2. Research Design and Methods

### 2.1. Study Participants

Participants were recruited in sequence at the National Endocrinology and Metabolism Center (Level iii) of a University Hospital in Nanjing, China, from March 2021 to February 2022. The study inclusion criteria were: a diagnosis of type 1 or type 2 diabetes mellitus; having been treated with insulin injections for at least the last 6 months; and no extended cutaneous diseases. The exclusion criteria were: being prescribed a glucagon-like peptide-1 agonist; having dermatitis or any other cutaneous disease; and having other diseases that may affect HbA1c, such as anemia. This study was conducted in accordance with the Declaration of Helsinki. Approval for the study protocol was granted by the research ethics committee of the First Affiliated Hospital of Nanjing Medical University (2019-SR-268). Patients were informed about the aim and methods of the study verbally and in written form. We obtained written informed consent from all participants before enrollment in the study.

### 2.2. Data Collection

In this study, trained medical professionals consulted the medical records of enrolled patients, and extracted the following information: age, gender, body mass index, type of diabetes, diabetes duration, insulin injection duration. After that, all the extracted information was verified again by the professional with the patient to ensure the accuracy of the data. All of the personal information was recorded and kept confidential. 

Assessment of LHs: All participants underwent ultrasound scanning conducted by two qualified radiologists, respectively, following the unified protocol which has been reported elsewhere [5,15]. An Esaote My Lab60 linear multifrequency probe (6–18 MHz) was used for ultrasound scanning. Each patient underwent an additional examination at one centimeter above the navel (where insulin has never been injected) as their self-control. At each site, thickness, echo, the blood flow of subcutaneous tissue, and the boundary between the subcutaneous tissue and the dermis were examined and recorded carefully to find out whether the patients had LH according to the criteria described by Kapeluto et al. [6,16]. If the results of the two radiologists were inconsistent, a third radiologist helped to make the final judgment. The inter-examiner agreement was high, with a Cohen’s kappa coefficient of 0.95.

If different types of LH were found in a same patient, we later classified the patient into the subgroup of the most serious type of LH found by the patient.

LHs follow-up at six months: In order to observe the ultrasonic changes of LHs, 10 patients with nodular LH, 5 with diffuse LH, and 2 with hypoechoic LH were randomly selected for follow-up 6 months later. The LH sites of these patients were specifically marked and photographed. The researchers and patients kept the body surface photo of the lesion part, respectively. The patient was told to refer to the photo before each insulin injection to make sure that the lesion site was not injected any more. Meanwhile, the researchers needed to ensure that the previous LH locations were correctly identified at follow-up according to the detailed records and photographs.

Assessment of HbA_1C_: Two milliliters of whole blood was taken from all patients and placed in an anticoagulant EDTA test tube, and HbA1c was detected via a Clover A1c analyzer (D10 hemoglobin detection system Specifications).

All participants diagnosed with LH by ultrasound were informed of the specific extent and location of any LH areas and advised to avoid further insulin injections into these areas. Doctors were also advised to adjust insulin doses to reduce the risk of hypoglycemia when injected at sites without LH.

### 2.3. Statistical Analysis

Continuous variables were reported as mean ± SD or *M* (Q1,Q3), and categorical variables were summarized as rate or percentage. Independent sample *t*-tests and one-way ANOVA were used to compare the data between groups. Odds ratios (ORs) with 95% confidence intervals (CIs) were used to report results. All data were analyzed using SPSS (version 26.0; SPSS Inc., Chicago, IL, USA). A *p*-value below 0.05 (*p* < 0.05) was considered statistically significant.

## 3. Results

### 3.1. Sample Characteristics

In total, 344 patients were enrolled in this study. Among them, 195 patients (56.7%) were male. The study population had a median age of 57 (30, 66) years. Of the 344 participants, 136 had type 1 diabetes and 208 had type 2 diabetes. The median insulin exposure duration is 6.2 (2.8, 11.0) years. Their average BMI was 23.3 ± 4.0 kg/m^2^.

### 3.2. Lipohypertrophy Findings and Characteristics

**Figure 1:**
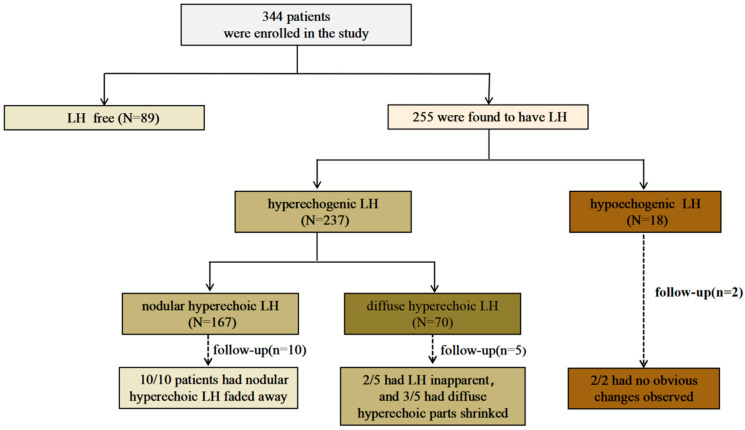
Lipohypertrophy findings in this study.

Of all 344 patients, 255 (76.1%) were found to have LH via ultrasound. The LH imaging manifestations of these 255 patients were mainly classified into two types, hyperechoic LH (237 cases) and hypoechogenic LH (18 cases). Of all patients with hyperechoic LH, 167 showed nodular hyperechoic LH, and 70 showed diffuse hyperechoic LH. (Figure 1).

**Figure 2:**
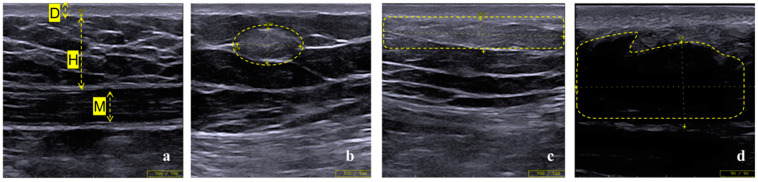
Imaging features of normal tissue and LH under ultrasound. (**a**) D, dermis; H, hypodermis (mainly adipose tissue); M, muscular layer. Each layer is clearly demarcated. (**b**) Nodular hyperechoic LH with obvious space occupying sensation. (**c**) Diffuse hyperechoic LH presented with no clear demarcation from the dermis. (**d**) Hypoechoic LH with irregular boundary, which is similar to adipose tissue liquefaction.

Features of different types of LH examined via ultrasound are shown in Figure 2. The layers of skin and muscle are clearly demarcated in areas where insulin has never been injected, and the ultrasound heterogeneity in each layer is small (Figure 2a). Nodular hyperechoic LH refers to the nodular region with an abnormally increased echo in the subcutaneous fat layer in the insulin injection area, with relatively clear and measurable boundaries and no obvious envelope (Figure 2b). In contrast to nodular LH, diffuse hyperechoic LH is an abnormally hyperechoic region with no distinct boundaries in length and/or width (usually more than the field of view of one probe—the probe in this study provides a 40 mm diameter field of view), and in general, their depth can be measured (Figure 2c). Hypoechoic LH is characterized by extremely low echo or even no echo in the subcutaneous adipose tissue, and the fibrotic tissue within this area is almost invisible compared to the normal area (Figure 2d).

### 3.3. LH Follow-Up by Ultrasound Assessment

**Figure 3:**
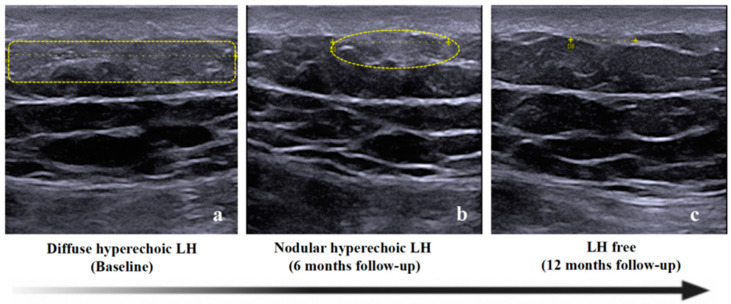
Examples of the changes in hyperechoic LH under ultrasound at follow-up. Diffuse hyperechoic LH at baseline (**a**) had obviously shrunk to nodular LH at 6 months follow-up (**b**). Additionally, the hyperechoic area could not be observed at 12 months follow-up (**c**) in the same patient (patient NO. 14).

**Figure 4:**
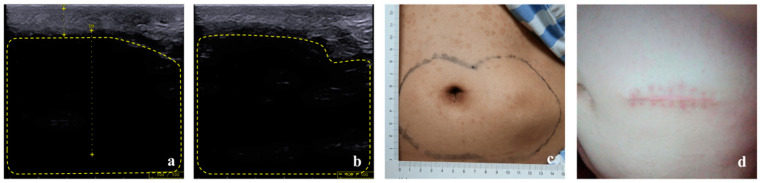
No obvious hints of improvement were observed in the case with hypoechoic LH at 6 months follow-up (**b** vs. **a**). The patient finally had his lesion removed by surgery (**d** vs. **c**). After stopping insulin injection at the LH lesion site, his required daily dosage of insulin decreased significantly (60 U vs. 20 U).

All 17 patients selected for follow-up reconfirmed that they had avoided the LH site for insulin injection in the past 6 months and had adopted the correct injection method. No newly emerged LH was found. At the follow-up after six months, obvious changes were observed in LHs of both hyperechoic types. All 10 patients with nodular hyperechoic LH displayed LH which had faded away. Of the five patients with diffuse hyperechoic LH, two had inapparent LH, and three had diffuse hyperechoic parts which shrank to nodular hyperechoic LH and then regressed. (Figure 3). The shrunken parts looked similar to the nodular LHs, with a slightly less regular boundary. No obvious changes were observed in two cases of hypoechoic LH. One patient ultimately chose minimally invasive surgery to remove the lesion (Figure 4).

### 3.4. Hypothesis on the Progression of Insulin Induced Lipohypertrophy

**Figure 5:**
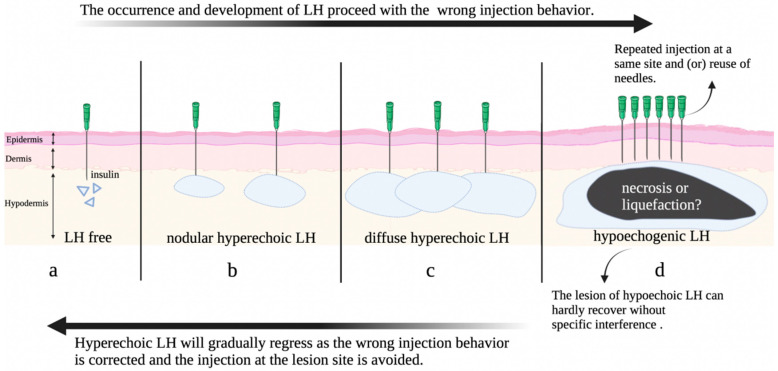
A hypothesis on the progression and revision of insulin induced lipohypertrophy.

It is certain that the layering of normal skin should be very clear (Figure 5a). Insulin needs to be injected into the normal hypodermis layer to exert its full effect on lowering blood sugar. Based on our clinical observations and the findings from this study, it could be hypothesized that the earlier stage of LH is nodular hyperechoic LH (Figure 5b). If nodular LH is not found in time and the patient continues to inject insulin at the LH site and/or reuse needles, LH will develop into a diffuse type (Figure 5c) or, even worse, a hypoechoic one (Figure 5d). Usually at this time, the dermis layer will also have obvious thickness changes. By strengthening the intervention of correct injection behavior, hyperechoic LH can gradually be repaired, but hypoechoic LH cannot easily heal by itself.

### 3.5. Differences of Blood Glucose Control in Patients with Different Types of LH

**Figure 6:**
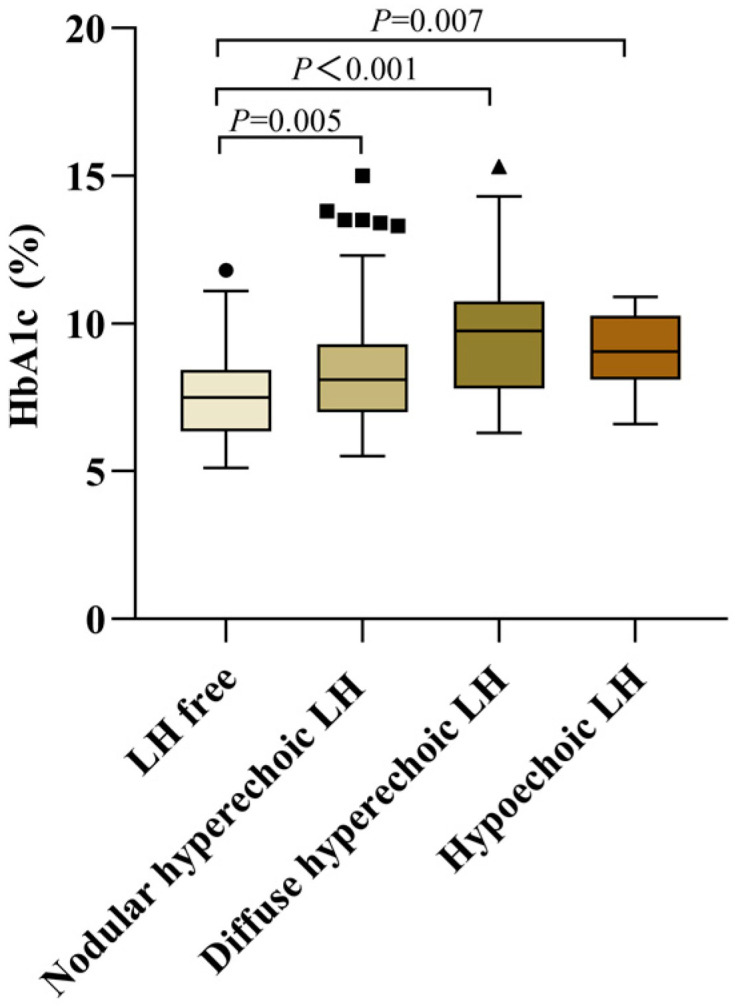
Comparison of HbA1c in different subtypes of LH (*N* = 344).

In order to further understand the effects of different types of LH on blood glucose and verify our hypothesis, all patients were tested for HbA1c at the same time of LH examination. Of all the patients, the most common type of LH observed was nodular hyperechoic LH (with mean HbA_1C_ 8.4 ± 1.9% (68 ± 20 mmol/mol)), followed by diffuse hyperechoic LH (with mean HbA_1C_ 9.6 ± 2.0% (81 ± 21 mmol/mol)), then hypoechoic LH (with mean HbA_1C_ 9.1 ± 1.3% (75 ± 14 mmol/mol)) (Figure 6). Patients with LH had significantly higher HbA_1C_ than those without (8.7 ± 1.9% vs. 7.6 ± 1.5% (72 ± 21 mmol/mol vs. 60 ± 17 mmol/mol), *p* < 0.001). On this basis, further group differences were detected using one-way ANOVA with Bonferroni post hoc tests. The test results showed that compared with the LH-free group, the mean HbA_1C_ of the nodular hyperechoic LH group increased by 0.8% (9 mmol/mol) (95% CI:−1.394~−0.168, *p* = 0.005), that of the diffuse hyperechoic LH group increased by 2.0% (21 mmol/mol) (95% CI: −2.696~−1.20, *p* < 0.001), and that of the hypoechoic LH group increased by 1.5% (16 mmol/mol) (95% CI: −2.689~−0.275, *p* = 0.007); compared with the diffuse LH hyperechoic LH group, the mean HbA_1C_ of the nodular hyperechoic LH group decreased by 1.2% (13 mmol/mol) (*p* < 0.001).

**Figure 7:**
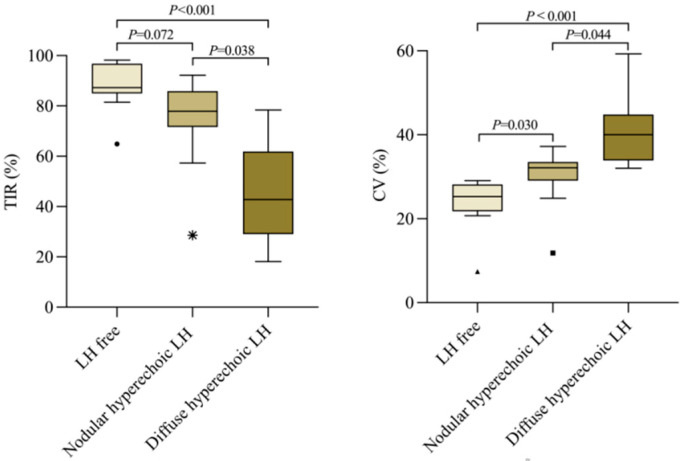
Comparison of TIR and CV in different subtypes of LH in T1DM patients (*N* = 34) * indicates abnormal values.

In addition, there were 34 patients with type 1 diabetes (T1DM) who had been wearing their flash glucose monitoring system (FGM) for more than 1 week when they were recruited to this study. After obtaining their consent, the original blood glucose values recorded by the system were derived. TIR and CV, as the two key indicators of blood glucose control, were calculated by using the calculation software Easy GV version 9.0 R from Oxford University (the data for the first 3 days were excluded considering the possible errors during the initialization of FGM). Ten of these 34 patients with type 1 diabetes were LH-free, 15 were with nodular hyperechoic LH, 9 had diffuse hyperechoic LH, and none had hypoechoic LH. Group differences of TIR and CV were detected using one-way ANOVA with Bonferroni post hoc tests. The results showed that the LH-free group had the highest TIR and the lowest CV, whilst diffuse hyperechoic LH had the lowest TIR and the highest CV among the three groups (Figure 7).

## 4. Discussion

Ultrasonography has been widely used in different clinical areas, especially in intensive care units [17]. At the same time, ultrasound technology has certain advantages in the differential diagnosis of certain diseases [18]. LH is typically diagnosed via visual inspection and palpation in clinical practice because of its operability and convenience. With the widespread use of ultrasonography, specialists have placed high-frequency ultrasound scanning in a more advanced position in identifying LH. The incidence of LH based on ultrasound diagnosis is 14.5–86.5%, and the median incidence is 56.6% [19]. In our study, the incidence of LH was 74.1% (255/344), which was consistent with previous reports, indicating that LH is a fairly common complication in patients with insulin injections, which attracts our attention.

One advantage of using ultrasound scanning to diagnose LH is that it cannot only judge whether the patient has LH or not but can also further provide a more objective presentation, especially in that ultrasound can clarify the nature, characteristics, and size of lesion sites. Previous studies have described the ultrasound features of LH and proposed specific, reproducible criteria for the detection of LH [16]. It must be noted that due to repeated injections and inflammatory reactions, a hyperechogenic area might be a sign of fibrotic tissue, so careful discrimination is necessary. Our study screened 344 subjects for LH using the same diagnostic criteria and found that the most common type of LH was hyperechoic. Which can be divided into the nodular hyperechoic type and diffuse hyperechoic type. Additionally, the incidence of hypoechoic type was lowest in the Chinese population. As far as we know, this classification and the associated manifestations of LH have not been previously summarized and described in detail. Meanwhile, the incidence of different types of LH has not been reported before. We firmly believe that the different types, characteristics, and incidence of each type of LH described in this paper are an important supplement to previous studies.

On the one hand, the staging of many diseases is based on the imaging of ultrasound examination, so we also try to classify LH based on different imaging manifestations. According to our follow-up, nodular hyperechoic LHs are the easiest to regress, diffuse hyperechoic LHs will gradually shrink to nodular hyperechoic LHs and then regress, while hypoechoic LHs are the most difficult to heal. So, we firmly believe that the earlier stage of LH is nodular hyperechoic LH. As time goes on, LH will develop into a diffuse type or, even worse, a hypoechoic one.

On the other hand, it is also an important prerequisite to combine different types of LH with patients’ metabolic indicators for classification. HbA_1C_ is the most commonly used indicator to measure blood glucose control in patients, because it is believed to reflect average blood glucose levels over several months and has a strong predictive value for diabetes complications [20]. To our knowledge, there is no study comparing HbA_1C_ in patients with different types of LH. Our results showed that patients with diffuse hyperechoic LH had worse blood glucose control than nodular hyperechoic LH. A possible explanation for this might be that diffuse LH is more serious than the nodular type regarding the effect on blood glucose. Similarly, our TIR and CV data of 34 type 1 patients with FGM also proved this.

Since the number of patients with hypoechoic type LH was small in this study, we could not confidently confirm the relationship between this type of LH and patients’ glycemic control, and we need to further increase the sample size to clarify this point. According to our clinical experience, the hypoechoic type is the most serious type, causing large glycemic variability and frequent hypoglycemia in patients, which is in accordance with the case report of Gentile S [21]. Additionally, this is consistent with our observation that patients with hypoechoic LH do not have the highest HbA_1C_ among all of the participants. This may be because more frequent episodes of hypoglycemia lead to false-negative HbA1c results.

Some studies have paid attention to the grading of LH. Demir G [22] graded LH from 0 to 3 according to the inspection and palpation of the insulin injection site. Considering the limitations of LH severity assessment based only on the number of lesions, Ucieklak D [23] proposed a new LH severity scale considering both the number and size of LH lesions and categorized LH into four stages of advancement. Additionally, Hashem R [24] proposed a conceptual model of an LH grading system. However, the above grading methods did not refer to the metabolic outcomes of patients. In this study, it is relatively more objective to grade LH based on the abnormal ultrasonic echo of the subcutaneous fat layer of the patient and their blood glucose control indicators.

The pathophysiological mechanism of LH still remains unclear. Among various proposed mechanisms, it is widely accepted that LH is both a local effect caused by the reaction of adipocytes to the insulin injection and also the anabolic effect that insulin has on local adipocytes [25]. Many studies have found that LH is strongly associated with poor injection behavior [26,27], especially needle reuse and wrong injection site rotation. Additionally, standard injection technology training is essential in helping patients to reduce the occurrence of LH [28,29,30]. At the same time, what we all know is that insulin should be injected into undamaged skin and the subcutaneous fat layer to maintain its normal absorption [31]. So, for ethical reasons, when LHs were found, we informed the patient of correct injection methods and told them to avoid injection at the lesion site. Therefore, we could not observe the regression of LH in patients, and thus our hypothesis about LH progression is based on a reverse reasoning of the regression of existing LHs. This hypothesis still calls for further in-depth investigation. If possible, animal models may be recommended.

Our study further confirms the importance of timely LH screening for patients who need routine daily insulin injections, as early detection can help to avoid the deterioration of LH. China’s technical guidelines for diabetes drug injection [32] state that patients with long-term insulin injections should be screened for skin complications at least once a year. It is also emphasized that the frequency of LH detection should be increased in patients who have already developed LH. Additionally, patients should be advised not to inject into the lesion site until the doctor confirms that LH has completely subsided so as not to interfere with insulin absorption.

## 5. Limitation

The data for TIR and CV in this study were only from 34 type 1 diabetes patients who wore an FGM system, and the results still need to be confirmed by further large-scale research participants.

Although HbA_1C_ is currently the “gold standard” for the evaluation of mean blood glucose control, we still need to pay additional attention to the hypoglycemia caused by injection insulin into LH, as severe hypoglycemia is a remarkable burden for patients with diabetes and increases the risk of adverse clinical outcomes [33], especially for patients with hypoechoic LH, as we mentioned previously. The relationship between various types of LH and patients’ hypoglycemia or non-perceived hypoglycemia needs to be further strengthened in future studies.

## 6. Conclusions

This study once again confirmed the high prevalence of LH in patients with insulin injection and the effect of LH on blood glucose. The most important contribution of this study is to put forward a scientific hypothesis about the progress of LH according to the high-frequency ultrasound image characteristics of patients. Furthermore, the hypothesis was effectively verified when combined with blood glucose control indicators. We believe that the present study lays the groundwork for future research into the classification and grading of LH.

## Data Availability

Data are available from the corresponding author upon reasonable request.
